# Context Modulates Outcome of Perinatal Glucocorticoid Action in the Brain

**DOI:** 10.3389/fendo.2014.00100

**Published:** 2014-07-09

**Authors:** E. Ronald de Kloet, Sanne E. F. Claessens, Jiska Kentrop

**Affiliations:** ^1^Department of Medical Pharmacology, Leiden University Medical Center, Leiden University, Leiden, Netherlands; ^2^Department of Endocrinology and Metabolism, Leiden University Medical Center, Leiden, Netherlands

**Keywords:** preterm birth, stress, glucocorticoids, antenatal, post-natal, human, rodent, brain

## Abstract

Prematurely born infants may be at risk, because of inadequate maturation of tissues. If there are signs of preterm birth, it has become common practice therefore to treat either antenatally the mother or postnatally the infant with glucocorticoids to accelerate tissue development, particularly of the lung. However, this life-saving early glucocorticoid treatment was found to increase the risk of adverse outcome in later life. In one animal study, the authors reported a 25% shorter lifespan of rats treated as newborns with the synthetic glucocorticoid dexamethasone, but so far this finding has not been replicated. After a brief clinical introduction, we discuss studies in rodents designed to examine how perinatal glucocorticoid action affects the developing brain. It appears that the perinatal action of the glucocorticoid depends on the context and the timing as well as the type of administered steroid. The type of steroid is important because the endogenous glucocorticoids cortisol and corticosterone bind to two distinct receptor populations, i.e., mineralocorticoid and glucocorticoid receptors (GR), while synthetic glucocorticoids predominantly bind to the GR. In addition, if given antenatally hydrocortisone is inactivated in the placenta by 11β-HSD type 2, and dexamethasone is not. With respect to timing, the outcome of glucocorticoid effects is different in early vs. late phases of brain development. The context refers to the environmental input that can affect the susceptibility to glucocorticoid action in the newborn rodent brain; early handling of pups and maternal care obliterate effects of post-natal dexamethasone treatment. Context also refers to coping with environmental conditions in later life, for which the individual may have been programed epigenetically by early-life experience. This knowledge of determinants affecting the outcome of perinatal glucocorticoid exposure may have clinical implications for the treatment of prematurely born infants.

## Introduction

The endogenous glucocorticoids cortisol and corticosterone promote the development of various tissues and organ systems during development. However, in prematurely born infants this effect of the steroids on maturation is abrogated resulting in a life-threatening situation. Therefore, it has become common practice to administer synthetic glucocorticoids such as dexamethasone to accelerate maturation, particularly of lung tissue (1, 2). If given antenatally to the mother the mortality of the prematurely born infant is decreased. While the infants obviously benefit from perinatal glucocorticoids, the treatment reportedly can have negative implications for later life outcome, particularly with respect to dexamethasone or its analogs (3–5). This health risk at adulthood includes impaired cardiovascular and immune functions as well as deficits in motor and cognitive performance, altered emotional reactivity, and neuroendocrine regulations.

In this contribution, data from animal and human studies with respect to the perinatal (antenatal and post-natal) action of glucocorticoids are presented. The action of the naturally occurring glucocorticoids is mediated by mineralocorticoid receptors (NR3C2, MR) and glucocorticoid receptors (NR3C1, GR) (6, 7). These glucocorticoids, cortisol, and corticosterone (the latter steroid only in rodents), bind to MR and GR with an order of magnitude difference in binding affinity (6). This implies that corticosterone and cortisol bind to the brain MR with high affinity resulting in substantial occupation even during the very low circulating hormone levels that are typically observed during the circadian trough and in rodents during the early post-natal stress-hyporesponsive-period (SHRP). These same steroids bind to GR only in adequate amounts when in adult animals their concentration has increased after stress and at the circadian peak. The membrane variants of MR and GR also display a lower affinity suggesting that their rapid effects preferably occur only at rising and high steroid concentrations during stressful situations and circadian peaks (8). No data exist on the role of these membrane variants during development. The synthetic glucocorticoids preferentially bind to GR, usually with high affinity (9).

We first review the human studies on antenatal activity of the hypothalamic–pituitary–adrenal (HPA)-axis, development of corticosteroid receptors and the effect of antenatal glucocorticoid treatment. Then, after an overview of antenatal rodent data, we will discuss the action of dexamethasone during the first post-natal days (pnds) of the newborn rodent, since this resembles most the antenatal treatment protocol in the human. Most animal studies have been performed with male animals; if females are used this is indicated. This contribution is concluded with a translational perspective that may be helpful in the clinical management of perinatal glucocorticoid treatment.

## Human Studies

### Antenatal HPA-axis development

During pregnancy, the activity of the maternal HPA-axis gradually increases over time toward a state of hypercortisolism that is caused by the steadily increasing production of corticotropin-releasing hormone (CRH) in the placenta and fetal membranes (10–12)). Fetal levels of CRH, adrenocorticotropic hormone (ACTH), and cortisol also rise, but not as much as the maternal values (13). Most of the cortisol present in amniotic fluid has a maternal origin and has successfully traversed the placenta. However, this accounts for only 10–20% of maternal cortisol because the remaining 80–90% is converted to inactive cortisone by the enzyme 11β-hydroxysteroid dehydrogenase type 2 (11β-HSD2) to protect the fetus from excess glucocorticoid exposure (14, 15). During the third trimester, fetal 11β-HSD2 levels fall and the fetus is exposed to high levels of CRH and endogenous cortisol (16). In this period, cortisol is able to exert its positive influence on the maturation of several organ systems, in preparation of survival after birth (17, 18).

### Antenatal MR and GR expression

Very limited information is available on human MR and GR expression patterns during gestation. Condon and colleagues reported minimal expression of MR and GR in peripheral tissues during gestational week 6, which becomes detectable from gestational weeks 8–16, with in general higher GR compared to MR expression (19). Noorlander et al. (20) studied MR and GR mRNA expression in the hippocampus from gestational week 24–34. They reported that both MR and GR mRNA are present during this period, with higher MR compared to GR mRNA levels and little change in expression levels over time (20). However, humans are in part altricial species and antenatal MR and GR expression appear to follow a pattern similar to that in guinea pigs and sheep rather than mice and rats. When comparing rodent brain development to human brain development, the last days of rat gestation (day 22–23) resemble human fetal brain development in week 16–17 regarding relative aspects of neurogenesis, neuron size, and number (21, 22).

### Antenatal glucocorticoids in the clinic

In 1972, Liggins and Howie were the first to demonstrate the beneficial effects of antenatal synthetic glucocorticoid treatment in the prevention of respiratory distress syndrome of premature infants (23). It is now known that treatment with the synthetic glucocorticoids dexamethasone and betamethasone accelerates the maturation process of several organ systems (e.g., lungs, heart, and kidneys) and antenatal synthetic glucocorticoid has become the standard treatment for women at risk of preterm delivery (1). In short-term, antenatal synthetic glucocorticoids reduce neonatal death, incidence of respiratory distress syndrome, cerebroventricular hemorrhage, necrotizing enterocolitis, respiratory support, intensive care admissions, and systemic infections in the first 48 h of life (2).

There is not much information on the long-term consequences of antenatal synthetic glucocorticoid treatment in humans. Roberts and Dalziel reported in a 2006 Cochrane systematic review, four follow-up studies into childhood and two into adulthood on the effect of a single course of antenatal glucocorticoid treatment (2). No psychological differences were found between antenatal glucocorticoid treatment and placebo for both children and adults. Another Cochrane review failed to show any long-term (adverse) effects of either single or repeated doses of antenatal betamethasone (24).

Despite this positive news on the clinical outcome of perinatal synthetic glucocorticoid treatment in the short-term, there are concerns about the potential negative long-term effects of antenatal overexposure. This is because of data from animal experiments with these synthetic glucocorticoids or derived from endogenous glucocorticoid action during stress (see below). Epidemiological studies have shown an association between high maternal stress, nutritional factors, or infections during pregnancy, which suggested an association of perinatal overexposure to endogenous glucocorticoid with mental disorders in later life. These include retrospective studies in man, such as those involving children born from Dutch mothers that were pregnant during the Dutch “Hunger winter” of 1944–1945. The adults exposed as fetus to these major stressors showed a higher incidence of schizophrenia (25), affective disorder (26), and addiction (27) as well as metabolic disorders (28). Furthermore, depression was associated with maternal influenza infection (29) and autism with mothers suffering from family problems (30). One review focused on animal models derived from epidemiological studies to examine the antenatal influence of maternal stress and nutritional status (31). In a meta-analysis of 60 human and 43 animal studies Beydoun and Saftlas (32) concluded independent effects of antenatal and post-natal outcomes. A vast literature is emerging on the outcome of early post-natal adversity and abuse (33, 34). Attempts are under way to ameliorate outcome using micronutrient supplements (35).

However, the consequences of treatment with the synthetic glucocorticoids betamethasone and dexamethasone cannot be directly compared to the impact of exposure to the endogenous glucocorticoids cortisol and corticosterone. First, the synthetic glucocorticoids are poor substrates for 11β-HSD2 and can pass the placental barrier more readily than endogenous glucocorticoids (36, 37). Second, the synthetic glucocorticoids bind the GR with higher affinity than endogenous glucocorticoids (9). Third, the synthetic glucocorticoids are GR-specific rather than MR-specific, while cortisol and corticosterone bind with a 10-fold higher affinity to MR compared to GR (6). And fourth, in the guinea pig MR and GR both are involved in the induction of multidrug resistance P-glycoprotein (P-gp) in the fetal blood–brain-barrier in response to endogenous cortisol, aldosterone, and dexamethasone (38).This finding suggests that upon repeated exposure to dexamethasone access of the synthetic steroid itself may be hampered, because the steroid is a substrate for the transporter (39–41). Regardless, the overall picture clearly indicates that glucocorticoid overexposure has long-term consequences and the question is therefore rather how these effects occur.

## Animal Studies

### Antenatal MR and GR expression

The programing effects of glucocorticoids during fetal brain development depend on the expression of MR and GR, which are time, location, and species specific. A distinction can be made between altricial and precocial species. Rats and mice are altricial species, which are relatively underdeveloped when born, with critical brain development occurring ante- and postnatally. In precocial species such as guinea pigs and sheep critical brain development occurs mostly antenatal. Primates including humans are considered a mixture of altricial and precocial: in aspects of body development they may be considered precocial, but behaviorally they are altricial.

In rats, GR mRNA expression was found in e.g., hippocampus, hypothalamus, cerebellum, raphe nuclei, locus coeruleus, and olfactory bulb, in midgestation from gestational day E12.5 onward (16, 42). At the end of the gestational period, when endogenous levels of corticosterone are high and the fetal HPA-axis becomes active, GR mRNA expression increases throughout the brain. MR mRNA expression is more limited in the hippocampus, parts of the hypothalamus and the superior colliculus piriform cortex, lateral septum, brainstem, and pituitary and starts in late gestation, 3 days before birth (16). A similar expression pattern was seen in mice (43–45).

In precocial species such as the guinea pig and sheep, a different and more complex MR and GR expression pattern was found (46–48). In guinea pigs, from gestational day 40 onward both MR and GR mRNA is present in the cortex, hippocampus, and dentate gyrus. In the period between gestational day 40 and 50, which is 60–75% of the total gestation length, GR mRNA levels increase while MR mRNA levels decrease. GR mRNA in the hippocampus increases to peak levels near term, while MR mRNA levels remain consistently low. However, in the paraventricular nucleus (PVN), GR mRNA levels are at peak level around gestational day 40 and 50 and show a large decline afterward (48).

### Animal models of antenatal glucocorticoid exposure

Many experimental animal studies have demonstrated that glucocorticoid overexposure *in utero*, both endogenous and exogenous, can have long-term effects on the offspring. Studies in rodents have shown a relationship between antenatal glucocorticoid overexposure and signs of schizophrenia and depression, increased anxiety and impaired learning and memory [see review in Ref. (49)]. Antenatal glucocorticoid exposure in rats, guinea pigs, and non-human primates caused permanently elevated baseline corticosteroid levels and increased corticosteroid levels in response to stress (50–53). Excess antenatal glucocorticoids are also capable of permanently altering MR and GR expression in multiple brain areas (54–56) and can influence entire neurotransmitter systems (49, 57). Experimental animal studies in comparison to studies in man are far better capable of controlling variables, yet there are many differences in methodology that can influence the outcome, such as the type of stressor or synthetic glucocorticoid used and timing and duration of exposure. Additionally, the effects of glucocorticoid overexposure are also age, sex, and species specific, making it very difficult to translate the outcome of animal experiments to the clinic.

### Post-natal MR and GR expression

The ontogeny of the receptors has been carefully mapped over the years using different techniques such as receptor binding assays, immunocytochemistry, and *in situ* hybridization (58–62). In general, MR is abundantly expressed in limbic-cortical regions, such as hippocampus, septum, amygdala, and fronto-cortical regions that have a function in processing of stressful information. With all detection methods, MR density is found to be high already early postnatally in mice and rats and localized in the nucleus. Pituitary GR expression is also very high in early-life. In the brain, GR binding and mRNA expression gradually increase postnatally and at weaning have reached about 50% of its adult level in the rat, and already adult levels in the mouse.

Using immunocytochemistry, a profound change in translocation of GR was observed. Immediately after birth, high nuclear immunoreactivity (ir) of GR occurs widespread over the rodent brain, particularly in stress regulating centers such as PVN, the ascending biogenic amine neurons, hippocampus, and amygdala as well as fronto-cortical regions. Then, possibly because of the low circulating levels of corticosterone during the SHRP, nuclear GR-ir is very low before reappearing at around pnd 12. Interestingly, the initial high GR-ir in the hippocampal CA3 pyramidal neurons and the suprachiasmatic nucleus does not re-appear after pnd 7, suggesting that the function of the latter nucleus now is entrained by exposure to daylight rather than the circadian variation in glucocorticoids from the mother (63, 64).

MR and GR in hippocampus show a profound downregulation after prolonged maternal absence, while a procedure like handling can induce their expression (65–67). Interestingly, MR and GR density changes in parallel with the amount of maternal care the pup is exposed to, a change in receptor concentration that can be explained by the extent of methylation of the receptor gene promotor region (68, 69). In a classical experiment (70), infant rats were deprived of maternal contact for 24 h on pnd 3–4 and injected with saline or ACTH1-24 at the end of the deprivation period. They were then returned to their dams and weaned on pnd 21. At pnd 48, they were sacrificed (24 h post adrenalectomy) and the hippocampal MR and GR measured using an *in vitro* cytosol binding assay. Using this procedure in the male rats, deprivation and deprivation + ACTH resulted in a reduction of GR. MR was also significantly downregulated in the deprived males. In contrast, in the female rats, saline injections in deprived female rats resulted in increased GR capacity and ACTH injections lead to a further up-regulation of the GR. None of the early manipulations influenced the regulation of the MR in females. These results in adult (7-week-old) rats indicate that the corticosteroid receptor systems in the brain are sensitive to brief manipulations in maternal care as well as corticosterone levels occurring early in development. Moreover, there is a striking effect of sex.

### Animal models of endogenous post-natal glucocorticoid variation

In rodents, the first 2 weeks of life are characterized by a SHRP during which mild psychological stressors or exogenous ACTH, that trigger a pronounced corticosterone rise in adulthood, produce only a weak adrenocortical response (71, 72). The SHRP lasts from pnd 4 to 14 in rats, and from pnd 2 to 12 in mice (73). During the SHRP, circulating corticosterone levels are low and stable, but the steroid that circulates is free, since corticosteroid binding globulin (CBG) is not detectable during this period. Also metabolism of corticosterone is changed, and hence the pharmacodynamics of the hormone is different from that during the previous perinatal days as well as the postweaning period. Because of the lack of ACTH, the size of the adrenal is small and thus the capacity to secrete steroids is very low, demonstrating that adrenal hyporesponsiveness is actual the most proximal cause of the SHRP (74).

The mechanism underlying this period of adrenal quiescence has been examined thoroughly. It has been proposed that neural connections involved in processing of stressful information are still immature at the time. However, since adrenalectomy (ADX) during the SHRP triggers a large ACTH response, it seems that enhanced corticosterone feedback at the pituitary level is causal for the SHRP. Moreover, Schmidt et al. (75) demonstrated that the GR antagonist RU486, administered during the SHRP, triggered a profound ACTH and corticosterone response. Next, mice carrying a conditional knockout of the GR gene specifically targeted at the pituitary corticotrophs showed excessive amounts of circulating corticosterone during the SHRP (76). This elevated corticosterone was resistant to dexamethasone suppression suggesting that in the pup the pituitary is the primary feedback site of stress-induced HPA-axis activity. Indeed, a single exposure to a psychological stressor is capable to mount a central response in c-fos and CRH, but the adrenal remains quiescent under these conditions (77).

It is important to realize that GR-mediated feedback is in operation to maintain the SHRP and this phenomenon by definition only can be revealed in the context of a stressor. Hence, administration of RU486 in saline did not trigger a stress response and therefore no further disinhibition by the GR antagonist. If RU486 was administered in polyethylene glycol (PEG) as solvent, an immune response caused by inflammation at the injection site occurred that was sufficient to trigger adrenocortical corticosterone secretion revealing the effect of the antagonist (78).

Thus, during the SHRP, corticosterone inhibits the stress-induced HPA-axis activity primarily via a pituitary feedback site. The GR-mediated mechanism maintaining the SHRP can be revealed by pituitary GR knockout or GR antagonist treatment, but only if there is a corticosterone rise after stress. In case of resting conditions when GR blockade cannot operate because corticosterone levels are too low, the quiescence of the HPA-axis is maintained via a centrally driven MR mechanism. These MR’s operate brain circuits appraising salient information as they do during adulthood (78).

### Maternal care and not dexamethasone exerts long-term HPA-axis control

There is evidence that the effects of dexamethasone on the developing brain precede independent of psychosocial and attachment effects. The experiments were inspired by the seminal studies of Levine in the 1950’s demonstrating that daily handling of rat pups attenuated HPA-axis activity and emotional reactivity in adulthood, an effect that could be mimicked by enhanced maternal care (71, 72). MR and GR also respond; both handling and maternal care induced MR and GR, probably because of enhanced demethylation of the receptor gene (68, 79, 80). The opposite outcome was reached under conditions of prolonged maternal absence, a procedure that has become known as maternal deprivation.

**Figure 1:**
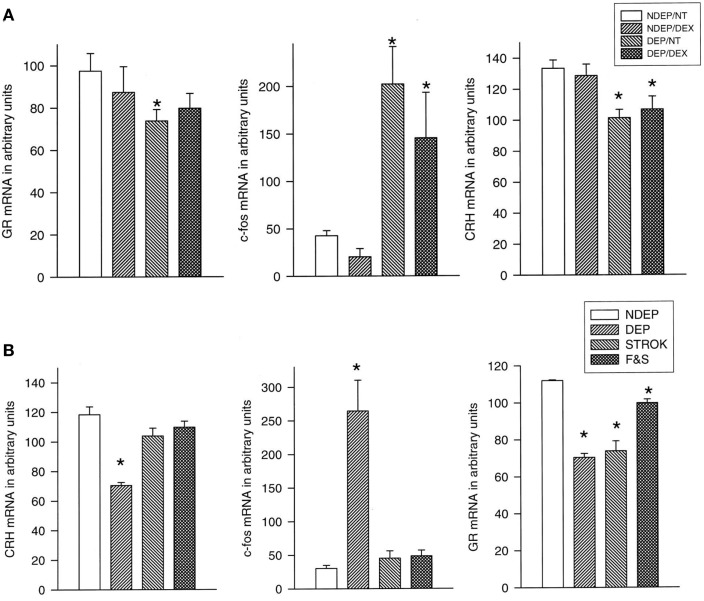
**Basal CRH stress-induced (30 min after saline injection) c-fos and basal GR mRNA expression in the PVN of 12-day-old pups**. **(A)** Effect of dexamethasone (DEX): the deprived (DEP) group had been deprived 24 h before testing (*n* = 10–12 per group). Non-deprived (NDEP) animals served as controls. The DEX animals had received a dexamethasone injection at the onset of deprivation (or equivalent time for NDEP) of 100 μg/kg BW. NT animals had received a saline injection instead of a DEX injection, *p < 0.05, significant from NDEP counterparts. **(B)** Effect of feeding and stroking: litters were deprived for 24 h on P11, during which time they were either left undisturbed (ISO), stroked (STROK), or stroked and episodically fed (F & S) (*n* = 10–12 per group). NDEP animals served as controls, *p < 0.05, significant from NDEP counterparts. [Reprinted with permission from Ref. (81)].

In response to 24 h of maternal deprivation of rat or mouse pups ACTH and corticosterone initially both rise during the first 8 h of maternal absence. Subsequently, the rise of corticosterone continues and reaches maximal levels after 24 h, while ACTH levels normalize for the remainder of the 24 h period. With the rise of corticosterone, the pituitary pro-opiomelanocortin (POMC) and CRH- and GR mRNA levels decrease in the PVN (66). If these 24-h deprived pups are exposed to an injection stressor a profound response occurs in pituitary ACTH and c-fos mRNA in the PVN. Amazingly, the pituitary ACTH and PVN c-fos response is normalized after stroking the pups for 45 s every 8 h with a warm wet artist brush in the anogenital region, mimicking maternal licking and grooming (81). For normalization of the corticosterone and the PVN GR mRNA response feeding is required in addition to stroking. Interestingly, dexamethasone (100 μg/kg) administration completely inhibits the ACTH and corticosterone response, but does not affect the central responses to maternal deprivation and the stressor (81) (Figure 1).

This differentiation between the effects of behavioral manipulations and the action of dexamethasone also persists. In a subsequent experiment, the rats deprived from pnd 11 to 12 for 24 h were pre-treated with dexamethasone to suppress the corticosterone response to maternal deprivation. Another group was only stroked for 45 s every 8 h or stroked + fed at that time, the latter procedure like dexamethasone also suppresses the deprivation-induced HPA-axis activity. At 20 days of age, the previously deprived + dexamethasone-treated animals did not differ in their endocrine and central responses. However, feeding and stroking did normalize the persistent effect of maternal deprivation on ACTH and corticosteroid receptors. Hence this study not only shows a lack of a persistent effect of dexamethasone, but also that the suppression of corticosterone by dexamethasone has no long-term consequences. It is the re-instatement of specific aspects of maternal care that counts in the long-term control of neuroendocrine function (82).

### Pups become used to repeated maternal separation

That the central components of the stress response are already in operation during the SHRP can be demonstrated also in another way. If the 24-h maternal deprivation at pnd 3–4 was split into three episodes of each 8 h per day from pnd 3 to 5, we observed during the first separation the predicted rise in ACTH and corticosterone secretion. These neuroendocrine responses disappeared with the next separations as if the pup has learned to anticipate the return of the dam. When the anti-glucocorticoid RU486 was administered the response to the first separation was further enhanced, but the effect of the antagonist declined in the second and was abolished after the third separation on pnd 5. In contrast, a MR antagonist decreased corticosterone levels after the first, but increased corticosterone secretion after the third separation. Surprisingly, while the newborn becomes used to repeated daily maternal separation as reflected by its normalization of basal HPA-axis activity to SHRP levels, the pups continue to respond to a novelty stressor (78, 83).

The importance of this information cannot be emphasized enough. It first shows that the pups as young as they are already have learned to cope with maternal absence. Their stress system stays on alert, however, and this process seems to involve the functioning of the MR. In the adult, this receptor has been shown to be important in controlling the circuits underlying the appraisal of salient events, vigilance, and emotional reactivity, and apparently this salience system already seems to be in operation in the newborn. In the adult amygdala, corticosterone is capable to impose metaplasticity in the amygdala (8, 84, 85). It appears that also in the pup the cooperative MR- and GR-mediated actions of corticosterone can enhance a lasting activity in the amygdala as can be read from the increased c-fos activity that persists into later life (86, 87). Whether glucocorticoids acting via GR are capable early postnatally to reallocate energy resources to circuits underlying executive function as they do in the adult, remains to be demonstrated (7, 88–90). That at this early age likely the salient rather than the fronto-cortical network operates also has become apparent from studies demonstrating the efficacy of the MR-dependent network in response to corticosterone during the SHRP (91).

In these experiments not only the duration of the separation leaves its marks, also the timing is relevant. If 24 h of maternal separation occurs at pnd 3–4, an enhanced stress-induced ACTH and hypothalamic c-fos response is observed at pnd 20. Deprivation for 24 h at pnd 11–12, however, results in the opposite, an attenuated ACTH and c-fos response to stress 8 days later at pnd 20 (92). The corticosterone response to stress in the pnd 20 pups deprived at either pnd 3 or 11 was not different from the non-deprived controls, however. Accordingly, the persistent alterations in central and ACTH responses as a function of pnd of deprivation are not reflected in corticosterone levels.

**Figure 2:**
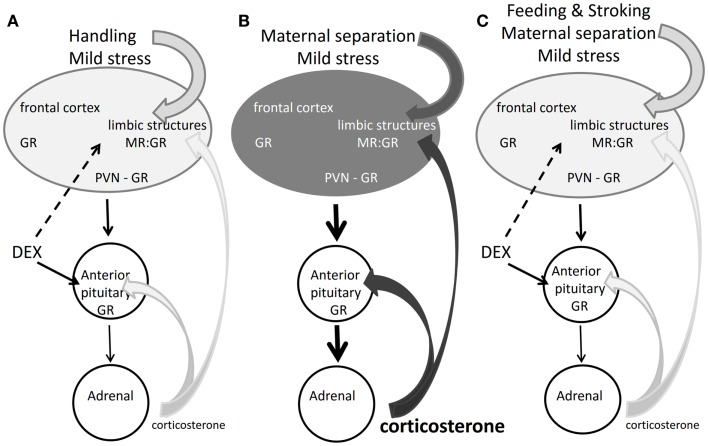
**Corticosterone, dexamethasone, and the rat SHRP**. **(A)** Mild stress + handling: corticosterone maintains quiescence in the HPA-axis, but the brain responds to the mild stressor. Dexamethasone acts on brain and pituitary via GR. Handling obliterates long-term effects of dexamethasone (93). **(B)** Maternal separation and mild stress: stressful information from the brain overrides the pituitary feedback action of corticosterone. The elevated corticosterone acts via MR and GR to modulate programing of limbic brain pathways (87). The darker gray tone indicates enhanced limbic activity by increased sensory and corticosterone input. **(C)** Feeding and stroking the maternally separated rat inhibits the brain, pituitary, and adrenal response to the stressor. Dexamethasone also blocks the pituitary–adrenal axis but does not give the same outcome as feeding and stroking (81).

In conclusion, the findings reported in the previous paragraphs demonstrate that post-natal dexamethasone effects are overridden by centrally regulated processes that underlie coping of the pup with changing conditions. The newborn is aware of these changing conditions and the corticosterone receptor system seems already to cooperate with the sympathetic nervous system in organizing the salience neuronal network response expressing signs of appraisal, attention, vigilance, and emotional reactivity. Why this network does not seem to be affected by dexamethasone may be because the synthetic steroid does not bind to the MR, which is one of the initial drivers of the salience network (Figure 2).

### Animal models of post-natal glucocorticoid exposure

Animal models of post-natal treatment with dexamethasone have demonstrated profound effects on later life outcome. Among the most serious outcomes is the finding that the lifespan of early dexamethasone-treated rats was reduced with 25% (94). The cause of death was cardiac and kidney failure, while also auto-immunity was noted. Impaired cognitive functions were also reported. The dexamethasone-treated animals had deficits in spatial learning and showed disturbances in the neuroendocrine response pattern to stress (95, 96).

**Figure 3:**
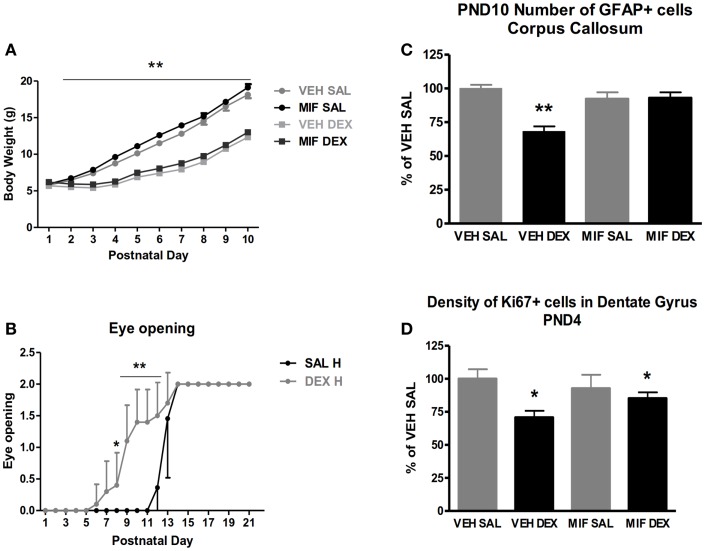
**Short-term effect of post-natal dexamethasone**. **(A)** Body weight on pnd 1–10 of saline (SAL) and dexamethasone (DEX) treated animals with intracerebroventricular (ICV) treatment of the glucocorticoid antagonist RU486 (mifepristone, MIF). DEX treatment significantly reduced body weight; this effect is not prevented by central mifepristone pre-treatment. **(B)** DEX treatment resulted in accelerated eye opening; on pnd 8–12 DEX treated animals show enhanced level of eye opening compared to SAL treated animals. Data represent mean ± SEM, *p < 0.05, **p < 0.01. **(C)** Number of glial fibrillary acidic protein (GFAP)-positive cells in the corpus callosum 7 days post treatment in SAL and DEX treated animals with ICV MIF pre-treatment. **Interaction between subcutaneous (SC) and ICV treatment *p* < 0.01. ^#^Interaction between SC and ICV treatment *p* < 0.10. **(D)** Density of Ki-67 positive cells in the dentate gyrus of the hippocampus 24 h post treatment in SAL and DEX treated animals with ICV MIF pre-treatment. **Main effect of DEX treatment *p* < 0.01; *main effect of DEX treatment *p* < 0.05. [Reprinted with permission from Ref. (97)].

We started a research project with the aim to ameliorate the putative negative prognosis of early dexamethasone treatment. In the first series of experiments, we have used an intracerebroventricular (ICV) administration of the anti-glucocorticoid RU486 to protect the brain against early dexamethasone treatment (97). We adopted the treatment regimen of tapering daily doses of dexamethasone-21-phosphate during the first 3 pnds, because it is thought to resemble the clinical administration of the drug: 0.5 μg/g pup on pnd 1 followed by 0.3 and 0.1 μg on pnd 2 and 3. The treatments were preceded by an ICV administration of 0.1 ng RU486. First, we examined with immunostaining for the Ki-67 marker the neuronal proliferation in the hippocampal dentate gyrus and found a profound reduction the day after dexamethasone treatment, which however was not inhibited by GR blockade. These effects were normalized by pnd 10. We also analyzed astrogliosis on the basis of glial fibrillary acidic protein (GFAP) immunocytochemistry and found a significant suppression in the corpus callosum 1 week after dexamethasone administration, that was blocked by pre-treatment with RU486 (Figure 3).

These data are consistent with previous studies showing that dexamethasone can transiently suppress neuronal proliferation and astrogliosis and that the effects are reversible possibly because of rebound effect later during development (98). The data also show that some of these effects can be prevented by local GR blockade in the hippocampus.

### Later life outcome

We were unable to reproduce the striking negative outcome of early post-natal dexamethasone treatment and discovered several factors that may account for this discrepancy with the published studies. First, in our studies we have used Long Evans rats, while most other studies were performed with Wistars or Sprague Dawley rats. More importantly, however, might have been differences in the design of the studies. While for instance Kamphuis et al. (96) used a whole litter design, we have consistently used a split-litter design with both dexamethasone and saline treated animals within every litter, to assure similar treatment conditions for each individual animal. Moreover, in the course of our experiments we noted that the handling procedure, required to inject and mark the pups, actually triggered bouts of enhanced maternal care that are known since long to reduce emotional reactivity and stress responsivity of the pups in later life. Indeed in a comparative study we found that a non-handled group of rats was more impaired in cognitive function than the handled dexamethasone and saline groups that each did not differ significantly from each other (93, 99).

Then in a controlled study, we examined whether daily handling actually could prevent adverse effects of post-natal dexamethasone treatment (93). Thus four groups were compared: first, a saline and a dexamethasone group that was only handled briefly for injections on the first 3 days of life and afterward was left undisturbed until weaning. Second, a saline and a dexamethasone group that was handled daily until weaning for 15 min. Eye opening was accelerated in dexamethasone-treated animals from day 12 to 8; a phenomenon that could not be evaluated in the non-handled groups. Dexamethasone treatment reduced body weight gain by 20% during the first 3 weeks of life. These differences in body weight lasted up to 10 months of age. Maternal care was enhanced the first week of life in the handled groups. This effect was only observed post-reunion when licking and grooming was increased from about 8 to 12% of the time. These differences in maternal care are known to have profound effects on later life outcome (100).

**Figure 4:**
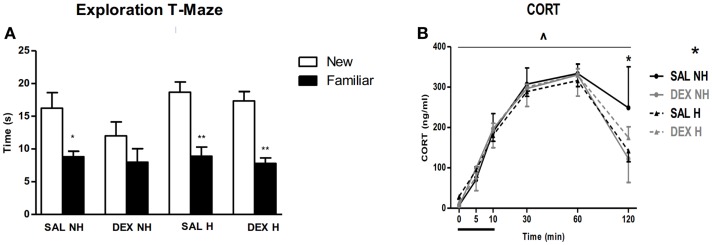
**Long-term outcome of post-natal dexamethasone**. **(A)** All groups except dexamethasone non-handled (DEX NH) spent significantly more time in the new compared to the familiar arm during re-exposure to T-maze. Data represent mean ± SEM, *p < 0.05, **p < 0.01. **(B)** Cortcosterone (CORT) levels before, during, and following exposure to 10 min restraint stress. Both handling and DEX treatment result in enhanced negative feedback of the HPA-axis at *t* = 120 min. Data represent mean ± SEM. Time × drug treatment × handling interaction *p* = 0.01, *p < 0.05 (from Ref. (93)).

A number of behavioral observations were made over the next 10 months. Handling improved impaired spatial learning of the dexamethasone-treated non-handled animals the T-maze (Figure 4). In another hippocampal-associated spatial learning test, the water maze, handling reduced the susceptibility to the impact of dexamethasone treatment. Furthermore, in a fear conditioning paradigm the acquisition of fear as expressed by a freezing response was reduced in both handling groups both immediately after the shock as well as upon re-exposure to the context after 24 h, without any effect of dexamethasone treatment, which is in agreement with Kamphuis et al. (95). Handling had a beneficial effect on pre-pulse inhibition at the 2 dB level in saline treated animals, without affecting dexamethasone-treated animals. Finally, both dexamethasone treatment and handling resulted in enhanced negative feedback of the stress-induced corticosterone response and reduced startle reactivity (Figure 4). Thus, we find that the handling procedure, which enhances maternal care received by the newborn, was able to attenuate specific dexamethasone-induced impairments in the behavioral phenotype of the adult animals (93).

### Developmental origin of disease

One may wonder how the outcome of early dexamethasone exposure may fit into the theory on the developmental origin of health and disease (DOHaD). This theory has its basis in the Barker hypothesis on the relationship between low birth weight and health outcome in later life and refers to the efficiency of food and energy transfer of the mother to the fetus (101). There can be many reasons for this deficit including the stressful events occurring antenatally and even the pre-implantation hormonal conditions (102). Ovarian hyperstimulation is a stressful condition that compromises vascularization of the blastocyst implantation site and hence caused decreased supply of nutrients. Ovarian hyperstimulation is commonly used in the generation of transgenic mice and might therefore have introduced a bias in all studies involving mutant mice (103, 104), particularly if urinary rather than recombinant gonadotropins have been used.

The DOHaD theory describes how experiences during early-life from blastocyst implantation, to fetal and post-natal life into puberty may induce developmental changes that affect the susceptibility to often comorbid cardiovascular and metabolic diseases as well as mental disorders in later life. It is thought that stressful experiences during early-life can modulate the genetic programing of specific brain circuits underlying emotional and cognitive aspects of behavioral adaptation. Although such a scenario implies multiple hits we have used for practical reasons to restrict the hits to three main categories: hit 1: genetic pre-disposition, hit 2: early-life experiences and hit 3: experiences during puberty (87, 105, 106).

### Cumulative stress exposure: The match/mismatch theory

It was found that either each subsequent hit accumulates damage and predisposes for disease or that gene X environment in early-life (hit 1 × hit 2 × hit 3) prepares for coping with challenges in later life. The latter situation predicts that experiencing adversity in early-life actually promotes coping with similar adverse conditions in later life. These findings led to the formulation of the predictive adaptive capacity or match/mismatch hypothesis (107). The latter hypothesis predicts increased vulnerability in case of a mismatch between early-life experiences that have re-programed the behavioral repertoire to cope with the actual circumstances the individual is facing in later life (108).

In our research, we observed that male rats that had experienced as pups abundant maternal care were well prepared at adulthood to execute cognitive tasks under relatively mild stressful conditions. Their hippocampus showed signs of extensive dendritic arborizations richly endowed by synaptic boutons and *in vitro* an efficient long-term potential (LTP) response could be evoked from these hippocampal circuits. However, under more stressful conditions this well-groomed offspring performed poorly, a finding that gives support to the match/mismatch hypothesis (100, 109–113).

What determines whether early-life adversity actually prepares for life ahead or for cumulative damage and enhanced vulnerability is not known. One line of research suggests that the genotype is critical. When newborn rats of a line that was genetically selected for enhanced responsiveness of the dopamine system by apomorphine-induced gnawing were deprived repeatedly from maternal care for a prolonged period, but only if the pups had also experienced a stressor (87). Subsequent exposure to isolation rearing during puberty promoted at adulthood signs of schizophrenia such as an impaired PPI response.

How early-life dexamethasone treatment fits into these hypothesis is not known. It is conceivable that the reduced body weight after pre- and post-natal dexamethasone exposure bears some relationship with the phenotype that led to the Barker and the DOHaD theories, but this needs to be investigated. At the same time, however, we also presented evidence that with respect to the brain the dexamethasone effects are overridden by maternal factors. This was demonstrated in two ways. First, in the deprived rat the normalization of HPA-axis activity persisted by mimicking maternal care through feeding and stroking the pups, but not if dexamethasone was given that also causes like maternal care an immediate suppression of the HPA axis. Second, adverse effects of dexamethasone on higher brain functions were ameliorated by neonatal handling causing enhanced maternal care.

## Mechanism

How do these maternal factors override the lasting effects of dexamethasone? Research in the past decade has clearly established that early-life experience may cause stable changes in histone acetylation and DNA methylation. These findings have led to the recognition of the epigenome as the molecular basis for altered plasticity in organ systems including the brain (114). These epigenomic signatures are a determinant of plasticity in neuronal networks underlying later emotional expression and cognitive performance, and seem a significant factor in the precipitation of stress-related mental disorders such as depression and addiction (115).

In recent years, DNA methylation of HPA-axis genes, among others the promotor regions of the GR (80, 116), the vasopressin (VP) gene (117), and 11βHSD (118) was observed. In particular, the epigenetic change in GR was found associated with a behavioral response pattern and HPA-axis setpoint in later life, and therefore is considered to represent some sort of “molecular memory” for salient events (68). Hence, decreased expression of GR in the hippocampus was found associated with emotional neglect in early-life (80, 119). Suderman et al. (116) recently stated that a global epigenetic signature of early-life experience becomes apparent that is maintained across species and centered around GR gene regulation, while more detailed analysis of the methylation profile may reveal more subtle intrinsic differences between species. Indeed, altered responsiveness in gene expression is maintained until adulthood and reflected in transcriptome analysis of subregions of the hippocampus (120). In this type of analysis it appeared already in the adult animals that the history of chronic stress experience causes a profound change in the transcriptional response to an acute corticosterone or stress stimulus with an overrepresentation of genes involved in chromatin remodeling, epigenetic processes, and cell adhesion (121–123).

The mechanism by which perinatal dexamethasone exposure causes long-term effects is not completely understood. Recent studies suggest that these long-term effects are possibly mediated through GR-activated epigenetic modulations such as DNA methylation and histone acetylation. Matthews and colleagues have extensively studied the epigenetic modifications associated with antenatal synthetic glucocorticoid treatment in the guinea pig. They observed that during the endogenous glucocorticoid surge in late gestation considerable changes in global DNA methylation in several organ systems take place that affect the expression of genes involved in the methylation process. Betamethasone administered before the natural glucocorticoid surge also affected global methylation and expression of methylation-related genes, though slightly different compared to the endogenous surge, and in addition affected histone-3 lysine 9 acetylation (124, 125). The effects of betamethasone treatment not only persisted into adulthood but also into the next generation (124).

In the male hippocampus it was also shown that concurrent with changes in DNA methylation, GR DNA binding was altered during both the endogenous glucocorticoid surge as well as synthetic glucocorticoid treatment (126). Though, this study clearly shows a relationship between altered GR DNA binding and epigenetic changes in gene expression, further research is necessary to provide conclusive evidence for a GR-mediated mechanism of epigenetic modification. This type of ChIP-seq analysis of GR binding to the hippocampal genome of ADX rats recently identified in the adult rat hippocampus 2460 significant binding sites of which 40% were associated with GRE’s in promoter regions (127).

## Concluding Remarks

This contribution was focused on the mode and mechanism of action in the brain of perinatal dexamethasone administration using pre- and post-natal animal models. This topic is of relevance because glucocorticoids are critical for survival of prematurely born infants. The glucocorticoid is required to ensure adequate maturation of in particular lung tissue. Regarding the mode of action the timing of glucocorticoids is important. If given preterm to the mother the synthetic glucocorticoids are used in a restricted number of courses [see Cochrane data base, (128)]. Hydrocortisone is ineffective preterm since the hormone is largely inactivated by 11β-HSD2 and does only poorly pass the placenta barrier. Postnatally hydrocortisone is used and reportedly there is so far no noticeable adverse outcome in later life (129). The underlying mechanism of the steroid effects depends on epigenetic modulation of the stress response system and possibly the changes in plasticity of limbic-forebrain glucocorticoid target regions.

The rodent is frequently used to examine adversity of dexamethasone treatment, because it is an altricial species and pnd 1 in the rat resembles the developmental stage at the beginning of the third trimester of human pregnancy. Alternatively, guinea pigs are used to mimic antenatal administration of dexamethasone to pregnant females. Here, we have discussed *in extenso* the role of glucocorticoids in development of the brain in relation to the HPA-axis. Some authors reported alarming effects of perinatal dexamethasone on health and brain development even causing a life shortening of 25% (94). However, we and others (130) were unable to find these detrimental effects, probably because our design included enhanced levels of maternal care. Using two different approaches, we demonstrated that such maternal care effects override dexamethasone effects in the brain and the HPA-axis. Hence, other approaches based on environmental input may be beneficial. For instance, in rats environmental enrichment during puberty appeared to neutralize the adverse outcome in the offspring of antenatally stressed pregnant dams (131).

Hence context is capable to modulate the outcome of perinatal glucocorticoid action. The context becomes manifest in two ways. First, maternal care and probably other contextual factors such as e.g., environmental enrichment are capable to modulate the outcome of glucocorticoid action, possibly by an epigenetic machinery. Second, context is equally important in later life where it either does or does not match the phenotype shaped by early experiences in sensitive windows of brain development during early post-natal life and puberty. Probably depending on genotype and epigenomic signature susceptibility to stress-related disorders may develop. Because of this interaction between dexamethasone-induced effects and experience-related factors, behavioral interventions appear to be crucial in the clinical management of preterm babies (132–134).

## Conflict of Interest Statement

E. Ronald de Kloet is on the Scientific Advisory Board and owns stock of Corcept Therapeutics, and also served on the Advisory Boards of Dynacorts Therapeutics and Pharmaseed Ltd. The other co-authors report no conflicts of interest.
